# Effect of Dapagliflozin on Myocardial Fibrosis After STEMI: A Double-Blind, Placebo-Controlled Randomized Trial

**DOI:** 10.3390/jcm15114061

**Published:** 2026-05-24

**Authors:** Luis Ortega-Paz, Claudio Laudani, Carlos Igor Morr, Alessandro Sionis, Pablo Vidal-Cales, Victor Arevalos, Rut Andrea, Oriol De Diego, Emilio Ortega, Francisco-Rafael Jimenez-Trinidad, Ana Paula Dantas, Dominick J. Angiolillo, Manel Sabaté, Jose T. Ortiz-Pérez, Salvatore Brugaletta

**Affiliations:** 1 Division of Cardiology, College of Medicine, University of Florida, Jacksonville, FL 32206, USAdominick.angiolillo@jax.ufl.edu (D.J.A.); 2 Division of Cardiology, Azienda Ospedaliero Universitaria Policlinico “G. Rodolico-San Marco”, University of Catania, 95123 Catania, Italy; 3 Cardiology Department, Cardiovascular Clinic Institute, Hospital Clínic Barcelona, University of Barcelona, 08036 Barcelona, Spaineortega1@clinic.cat (E.O.); masabate@clinic.cat (M.S.); 4 Department of Biomedical Sciences, Institut de Investigacions Biomèdiques August Pi i Sunyer (IDIBAPS), University of Barcelona, 08193 Barcelona, Spain; 5 Cardiological Intensive Care Unit, Cardiology Department, Hospital de la Santa Creu i Sant Pau, Sant Pau Biomedical Research Institute (IIB Sant Pau), 08041 Barcelona, Spain; 6 Department of Medicine, Autonomous University of Barcelona, 08193 Barcelona, Spain; 7 Division of Cardiology, Centro de Investigación en Red de Enfermedades Cardiovasculares (CIBERCV), 28028 Madrid, Spain; 8 División de Medicina Cardiovascular, Hospital de Clinicas, Universidad Nacional de Asunción, Asunción 2169, Paraguay; 9 Centro de Investigación Biomédica en Red de Fisiopatología de la Obesidad y Nutrición (CIBEROBN), Instituto de Salud Carlos III, 28028 Madrid, Spain; 10 Department of Cardiovascular Sciences, Fondazione Policlinico Universitario Agostino Gemelli—IRCCS, 00168 Roma, Italy; 11 Department of Pharmacology, Therapeutics and Toxicology, Universitat Autonoma de Barcelona (UAB), Cerdanyola del Valles, 08035 Barcelona, Spain

**Keywords:** dapagliflozin, myocardial fibrosis, ventricular function, myocardial infarction, sodium–glucose transporter 2 inhibitors, cardiac magnetic resonance, randomized controlled trial, ventricular remodeling, heart failure, extracellular space

## Abstract

**Background:** Myocardial fibrosis plays a key role in adverse remodeling after ST-segment-elevated myocardial infarction (STEMI). The effect of sodium–glucose cotransporter 2 inhibitors (SGLT2is) on myocardial fibrosis deposition among patients with STEMI undergoing primary percutaneous coronary intervention (pPCI) is unclear. **Objectives:** To assess the effects of SGLT2is on myocardial fibrosis among patients with STEMI undergoing pPCI. **Methods:** Patients with STEMI undergoing pPCI with left ventricular ejection fraction ≤ 50% were randomized to dapagliflozin 10 mg or placebo. The primary endpoint was cardiac magnetic resonance (CMR)-derived 6-month changes in remote myocardium extracellular volume (ECV) fraction from baseline. Secondary endpoints included changes in CMR-derived myocardial volumes, change in serum fibrosis biomarker levels, and adverse events. Multivariable adjustment for infarction location and diabetes status was performed as sensitivity. The study was halted prematurely due to slow recruitment. **Results:** Fifty-two patients underwent randomization between May 2021 and April 2024 and completed follow-up. At 6 months, dapagliflozin resulted in a non-significant reduction in ECV change compared to placebo (−0.39 [4.7] vs. 1.43 [5.7]; difference: −1.82 [−4.86; 1.23]; *p*-value = 0.235) while also leding to a higher degree of reduction in N-terminal pro-peptide of type III collagen (−177.0 pg/mL [416.1] vs. 3.6 pg/mL [553.8]; *p*-value = 0.208). No significant differences in other biomarkers or adverse events were noted in the main analysis. After adjustment, dapagliflozin was associated with increased reduction in left ventricular end-systolic volume (−4.02 mL [7.4] vs. 0.10 mL [10.1]; difference: −4.92 [−9.8; −0.1]; *p*-value = 0.047). **Conclusions:** In STEMI patients undergoing pPCI, dapagliflozin did not result in a significant reduction in ECV or biomarkers of fibrosis at 6 months.

## 1. Introduction

Myocardial fibrosis is the expansion of the cardiac interstitium due to the deposition of extracellular matrix proteins, and can be classified into diffuse interstitial fibrosis and focal replacement fibrosis [1]. Diffuse interstitial fibrosis involves the widespread deposition of collagen fibers between myocytes and is commonly observed in chronic conditions such as hypertension or heart failure [2]. Conversely, focal replacement fibrosis results from the deposition of scar tissue that replaces necrotic myocardial tissue, usually following events like myocardial infarction (MI) [2]. Notably, both diffuse and focal fibrosis contribute to ventricular dysfunction, and are associated with adverse long-term outcomes [2,3]. In patients with ST-segment elevation MI (STEMI), focal replacement fibrosis develops at the site of the infarction, while diffuse interstitial fibrosis affects remote myocardium, triggered by ischemia and subsequent inflammation [4].

Sodium–glucose cotransporter 2 inhibitors (SGLT2is) provide cardiovascular benefits beyond glucose control, including reductions in heart failure hospitalizations and cardiovascular mortality in patients with or without type 2 diabetes mellitus (T2DM) [5]. Preclinical studies have shown that SGLT2is modulate myocardial fibroblast activity by reducing fibroblast migration and collagen deposition [6]. Moreover, in individuals with T2DM and stable coronary artery disease (CAD), SGLT2is have been associated with a reduction in myocardial extracellular volume (ECV) measured by cardiac magnetic resonance (CMR), a marker of myocardial fibrosis [7]. However, the effects of SGLT2is in remote myocardial fibrosis and ventricular function in the acute phase after STEMI have not been studied.

The effect of DAPAgliflozin on myocardial fibrosis and ventricular function in patients with STEMI (DAPA-STEMI) trial sought to evaluate the impact of dapagliflozin, compared to placebo, on myocardial fibrosis and ventricular function in patients presenting with ST-segment elevation myocardial infarction, using CMR imaging and circulating biomarker assessment.

## 2. Methods

### 2.1. Trial Design and Oversight

The methods of the DAPA-STEMI trial (NCT06619600) were described previously [8]. Briefly, the DAPA-STEMI trial is a prospective, double-blind, placebo-controlled randomized trial conducted at Hospital Clínic and Hospital de la Santa Creu i Sant Pau in Barcelona, Spain. The trial evaluated the effects of dapagliflozin 10 mg daily on reducing myocardial fibrosis compared to placebo in patients with their first clinically diagnosed STEMI undergoing primary percutaneous coronary intervention (pPCI).

The trial protocol and subsequent amendments were conceived and designed by the Trial Steering Committee (Supplementary Materials) and approved by the Spanish Agency of Medicines and Medical Devices, as well as the Research Ethics Committee of each participating center. The trial was sponsored by the Spanish Society of Cardiology. Funding for the trial, as well as dapagliflozin and the matching placebo was provided by AstraZeneca (ESR-19-14489). The sponsor and funder had no role in the design or conduct of the trial and were not involved in data analysis or interpretation, nor in the redaction of this manuscript.

Two protocol amendments were made during the study period. The first amendment was made before starting enrollment to allow the inclusion of participants with and without T2DM, based on the results of the Study to Evaluate the Effect of Dapagliflozin on the Incidence of Worsening Heart Failure or Cardiovascular Death in Patients With Chronic Heart Failure (DAPA-HF) trial [9]. The second amendment, made after enrollment of 3 patients, allowed the inclusion of patients with a left ventricular ejection fraction (LVEF) of ≤50%, in light of the results of the Dapagliflozin Effects on Cardiometabolic Outcomes in Patients With an Acute Heart Attack (DAPA-MI) trial [10], and to also include patients with different locations than anterior MI. Additionally, the window for performing the baseline CMR was extended to up to 15 days after the index event. After 37 months from the study start date, the steering committee decided to terminate the study early due to slow enrollment.

An independent clinical events committee and core laboratory, both unaware of the trial-group assignments, adjudicated clinical, CMR, and laboratory outcomes (Supplementary Materials). Research staff at each participating site gathered the data. The authors had access to trial data and vouch for the completeness and accuracy of the data and adherence to the protocol. The co-first authors wrote the first draft of the manuscript, and all authors contributed to subsequent revisions and approved the final version for submission. The corresponding authors approved the final version before submission. The study was conducted in compliance with the protocol, the Declaration of Helsinki, and applicable local ethical requirements.

### 2.2. Participants

Patients between 30 and 85 years of age presenting with first clinically diagnosed STEMI undergoing pPCI were considered eligible for trial inclusion if they had a LVEF ≤ 50% and were hemodynamically stable (i.e., Killip class I) at the time of baseline CMR. Exclusion criteria included treatment with fibrinolytic therapy, previous treatment with SGLT2 inhibitors, type 1 diabetes mellitus, severe liver disease (Child-Pugh class C), chronic kidney disease defined as stage III or worse (estimated glomerular filtration rate [eGFR] < 45 mL/min/1.73 m^2^), systolic blood pressure < 90 mmHg at the screening visit, and any absolute contraindication to receive dapagliflozin or undergo CMR. A complete list of inclusion and exclusion criteria is provided in the Supplementary Materials.

### 2.3. Randomization and Blinding

The participants were randomly assigned in a 1:1 ratio to either receive dapagliflozin 10 mg daily or placebo within 24 h from the baseline CMR study. The randomization list was generated by an independent statistician using a computer-based program for stratified randomization, with presence of diabetes and infarction site (anterior vs. non-anterior) used as stratification variables. The trial pharmacist was the only person with access to the allocation codes throughout the whole study. The trial employed a quadruple-blind design to ensure that participants, care providers, outcome assessors, and data analysts were unaware of the treatment allocation. Emergency unblinding was allowed only when knowledge of the assigned intervention of the participant was essential for the management of a serious adverse event or a critical medical situation.

### 2.4. Trial Procedures

All participants received standard post-MI care in accordance with international guidelines, including beta-blockers, angiotensin-converting enzyme (ACE) inhibitors or angiotensin receptor blockers (ARBs), statins, and dual antiplatelet therapy [11]. Participation in routine cardiac rehabilitation programs was encouraged. After the end of the study, treatment decisions were made at the discretion of the treating physician for each participant.

Follow-up visits were performed at 1, 3, and 6 months, either in person or via telephone consultation when in-person visits were not feasible. Medication adherence was assessed by pill count at each follow-up visit. Outcome data, including CMR, were collected for participants who discontinued or deviated from the protocol, when feasible.

CMR was performed at baseline (i.e., within 15 days from the index event) and at 6 months following randomization using a 3T scanner (ARCHITECT, General Electric, Waukesha, WI, USA) equipped with a phased-array body surface coil and a cardiac-dedicated package with electrocardiogram gating. The imaging protocol included conventional steady-state free-precession cine images, T2 maps prior to contrast injection, T1 maps acquired before and 15 min after contrast injection, and late enhancement images acquired 10 min after contrast injection. The complete CMR acquisition protocol and complete list of CMR definitions are provided in the Supplementary Materials.

Venous blood samples for biomarker analysis were collected at 3 time points: baseline (i.e., after informed consent and prior to randomization), and at 3- and 6-month following randomization. A complete list of assessed biomarkers and used kits is provided in the Supplementary Materials.

### 2.5. Trial Outcomes

The primary endpoint was the change in ECV of the remote myocardium from baseline to 6 months, as measured by CMR. Secondary outcomes included changes in circulating biomarkers from baseline to 6 months, specifically C-terminal pro-peptide of type I procollagen (PICP), procollagen type III N-terminal peptide (PIIINP), galectin-3 (Gal-3), and high-sensitivity troponin I (hs-TnI).

Exploratory outcomes included changes from baseline to 6 months in anthropometric data (i.e., weight, abdominal circumference, and hip circumference), additional CMR measures of cardiac function and remodeling (i.e., LVEF, left ventricular end-diastolic volume [LVEDV], left ventricular end-systolic volume [LVESV], stroke volume [SV], left ventricular mass [LVM], infarct mass, and infarct size), and changes in other circulating biomarkers, including N-terminal pro-B-type natriuretic peptide (NT-proBNP) and suppression of tumorigenicity 2 (ST2). Safety measures included the incidence of death, MI, non-fatal stroke, heart failure hospitalization, revascularization, and acute liver failure. Data on any additional adverse reaction potentially related to the study medication were collected.

### 2.6. Statistical Analysis

Based on previous studies conducted by this research group [3,6], and assuming a change of 1.5% ± 2.2% in the ECV of the remote myocardium, measured by CMR, from baseline to six months in patients with STEMI receiving placebo, and an expected 85% relative reduction in the experimental group, we calculated that the inclusion of 94 participants (47 per group) would provide an 80% power to detect such a difference, with a two-sided alpha error of 0.05. To account for a 20% loss to follow-up and to ensure balanced stratification, the final sample size was set at 120 participants (60 per treatment group). However, the steering committee decided to stop enrollment prematurely after the enrollment of 54 patients due to slow recruitment.

The prespecified statistical analysis plan is reported in the Supplementary Materials. The primary endpoint was assessed using the mean difference and 95% confidence interval (CI) between the allocated treatments. Continuous variables were summarized as mean (standard deviation) or median (interquartile range), depending on the normality of distribution, and compared using the Student’s *t*-test or Mann–Whitney U test, as appropriate. Categorical variables were summarized as counts and percentage, and compared between groups using chi-square test or Fisher exact test, as appropriate. A mixed-effect model for repeated measures adjusted for the presence of T2DM and the localization of myocardial infarction (anterior vs. non-anterior) was used as a sensitivity analysis to assess the robustness of the results. All analyses were performed in the intention-to-treat population and repeated in the per-protocol as a sensitivity analysis.

All *p*-values were two-sided. A *p*-value < 0.05 was considered statistically significant for the primary endpoint, while other *p*-values were considered hypothesis-generating. All analyses were performed using R version 4.3.1 (R Foundation for Statistical Computing, Vienna, Austria) and SAS software, version 9.4 (SAS Institute Inc., Cary, NC, USA).

## 3. Results

### 3.1. Participants and Enrollment

**Figure 1:**
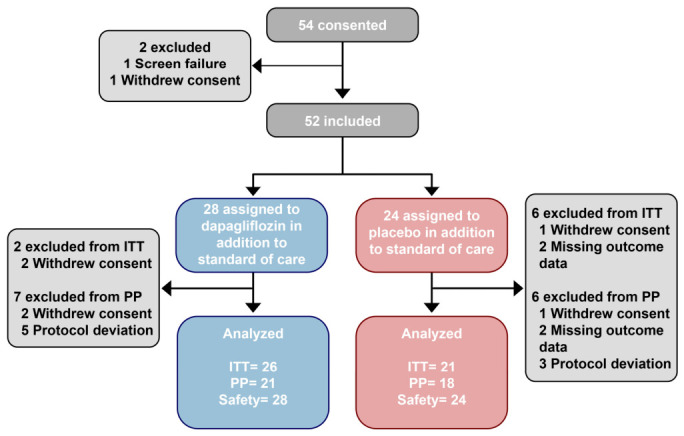
**Consort diagram of study flow.** The figures illustrate the flow of patients included in the DAPA-STEMI trial. Abbreviations: ITT, intention-to-treat PP, per-protocol.

From May 2021 to April 2024, a total of 54 patients were enrolled. Of these, one patient withdrew consent prior to randomization and one patient was excluded due to screening failure. Therefore, 52 patients were randomized, with 28 allocated to dapagliflozin and 24 to placebo (Figure 1), and all were included in the analysis.

Baseline characteristics were balanced between groups (Table 1). In the overall cohort, the mean age was 59.7 years; 49 patients (94.2%) were male, and 5 (9.6%) had T2DM. Procedural characteristics were generally balanced between groups (Supplementary Table S1), with 39 (75%) patients presenting with anterior MI, and the proximal left anterior descending artery being the more common culprit segment (23 patients, 44.2%). Medical management at discharge was similar, with a median hospital stay of 4.5 days (Supplementary Table S2). Diabetes management at baseline and discharge was similar between groups (Supplementary Table S3), as well as baseline CMR characteristics (Supplementary Table S4). The mean LVEF was 42.4% ± 5.0%, mean LVEDV 149 ± 34.5 mL, and mean infarct size 25.0 ± 15.0 g.

### 3.2. Primary Outcome

**Figure 2:**
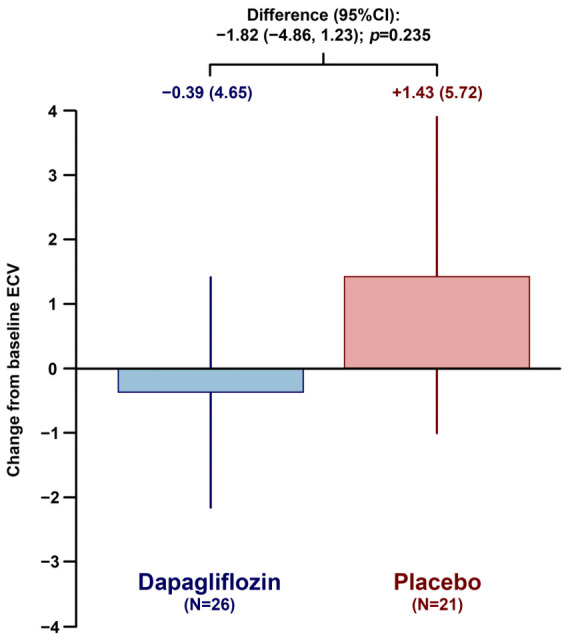
**Results for the primary endpoint.** The figure illustrates the comparison and treatment effect estimates in the dapagliflozin (blue) and placebo (red) groups. The change from baseline in each group is reported on top of each box, along with between-group differences. Abbreviations: CI, confidence interval; ECV, extracellular volume fraction remote; N, number of patients.

Among the 52 patients enrolled, 47 completed the 6-month CMR protocol and were included in the primary analysis. At 6 months, dapagliflozin was associated with a non-significant reduction in ECV change compared to placebo (−0.39% [4.7] vs. 1.43% [5.7]; difference: −1.82 [−4.86; 1.23]; *p* = 0.235; Table 2; Figure 2). The result was consistent after adjustment for T2DM status and localization of myocardial infarction (adjusted difference: −1.66 [−4.73; 1.41]; *p* = 0.281). The effects of dapagliflozin were generally consistent across prespecified subgroups, although a higher treatment effect was observed among patients with higher levels of galectin 3, but without significant between-group interaction (Supplementary Figure S1).

### 3.3. Secondary and Exploratory Outcomes

#### 3.3.1. Secondary Outcomes

**Figure 3:**
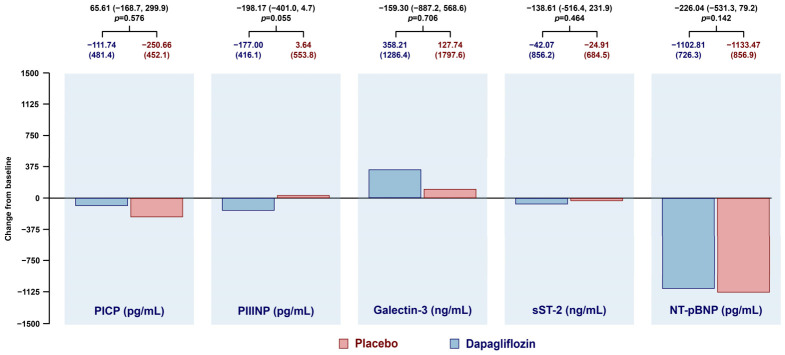
**Results for the biomarkers change from baseline to 6 months.** The figure illustrates the comparison and treatment effect estimates in the dapagliflozin (blue) and placebo (red) groups for each biomarker, highlighted at the bottom of each box. The change from baseline in each groups is reported on top of each box, along with between-group differences. Abbreviations: CI, confidence interval; NT-pBNP, N-terminal pro–B-type natriuretic peptide; PICP, C-terminal pro-peptide of type I procollagen; PIIINP, procollagen type III N-terminal peptide; sST-2, serum suppressor of tumorigenicity 2.

At 6 months, dapagliflozin led to a non-significant change from baseline in PIIINP compared to placebo (−177.0 pg/mL [416.1] vs. 3.6 pg/mL [553.8]; *p* = 0.055; Figure 3). Similarly, no significant differences were observed between groups for the change in PICP (−111.74 pg/mL [−481.4] vs. −250.66 pg/mL [−452.1]; *p* = 0.576), Gal-3 (358.21 ng/mL [1286.4] vs. 127.74 ng/mL [1797.6]; *p* = 0.706), or hs-TnI (−127,153.00 ng/dL [−113,073.0] vs. −219,529 ng/dL [189,545.4]; *p* = 0.363; Supplementary Table S5).

#### 3.3.2. Exploratory Outcomes

No significant differences were observed between groups for changes in NT-proBNP (−1102.81 pg/mL [726.3] vs. −1133.47 pg/mL [856.9]; *p* = 0.142) and sST2 (−42.07 ng/mL [856.2] vs. −24.91 ng/mL [684.5]; *p* = 0.464; Supplementary Table S5).

Among anthropometrical measures, dapagliflozin was associated with a significant reduction in abdominal circumference at 6 months, but without significant differences compared with placebo (−3.92 cm [−6.4, −1.5] vs. −0.31 cm [−3.5, 2.9]; difference: −3.66 [−7.84, 0.52]; *p* = 0.085). No other significant changes in anthropometric values were detected (Supplementary Table S6).

Among changes in CMR parameters, LVEF increased similarly in both groups from baseline to 6 months (6.11% [7.3] vs. 3.54% [7.5]), while LVM (−7.41 g/m^2^ [9.3] vs. −6.28 g/m^2^ [10.5]) and infarct mass (−4.33 g [10.4] vs. −6.71 g [11.8]) decreased to a similar extent in both groups (Table 2, Supplementary Table S7). No significant differences between groups were observed for additional CMR parameters (Table 2).

**Table 2:** Changes in cardiac magnetic resonance endpoints from baseline to 6-month follow-up.

	Total N = 47	DAPA N = 26	Placebo N = 21	*p*-Value
**Time between CMRs (months), median (IQR)**	6.2 (6.0, 6.5)	6.1 (6.0, 6.5)	6.2 (6.0, 6.5)	0.589
**Extracellular volume fraction remote**				
Baseline	26.87 (3.39)	27.10 (3.49)	26.59 (3.31)	
Months 6	27.29 (4.36)	26.71 (3.78)	28.02 (4.98)	
Change	0.42 (5.18)	−0.39 (4.65)	1.43 (5.72)	
Change difference		−1.82 (−4.86, 1.23)		0.235
Change (%)	2.81 (19.56)	−0.36 (16.82)	6.74 (22.30)	
Change difference (%)		−7.10 (−18.58, 4.40)		0.220
**Indexed extracellular volume fraction remote**				
Baseline	16.11 (4.0)	16.06 (3.8)	16.17 (4.2)	
Months 6	16.42 (4.4)	13.92 (4.1)	15.46 (4.1)	
Change	0.31 (0.3)	−2.23 (3.2)	−0.71 (4.8)	
Change difference		−1.52 (−3.80, 0.81)		0.185
Change (%)	3.16 (19.90)	−0.11 (17.30)	6.75 (22.30)	
Change difference (%)		−6.86 (−18.94, 5.23)		0.259
**Indexed intracellular compartment volume**				
Baseline	1550.95 (383)	1545.95 (381)	1556.42 (407)	
Months 6	1581.70 (431)	1533.45 (439)	1634.55 (438)	
Change	30.75 (316)	−12.50 (285)	78.10 (347)	
Change difference		−90.62 (−283.15, 101.90)		0.348
Change (%)	3.34 (20.70)	−0.07 (18.00)	7.07 (23.20)	
Change difference (%)		−7.15 (−19.73, 5.44)		0.258
**Left ventricular ejection fraction**				
Baseline	42.43 (5.0)	41.53 (5.6)	43.49 (3.7)	
Months 6	47.32 (9.6)	47.64 (9.5)	47.04 (9.5)	
Change	4.70 (10.2)	6.11 (7.3)	3.54 (7.5)	
Change difference	4.82 (−1.12, 10.76)			0.110
**Indexed left ventricle end diastolic volume**				
Baseline	76.82 (16.6)	77.09 (14.6)	76.48 (19.1)	
Months 6	78.01 (20.3)	78.27 (18.4)	77.69 (22.9)	
Change	1.19 (10.5)	1.18 (11.8)	1.21 (9.0)	
Change difference	−0.03 (−6.3, 6.3)			0.993
**Indexed left ventricle end systolic volume**				
Baseline	43.83 (10.1)	44.95 (8.3)	42.44 (12.1)	
Months 6	41.65 (15.5)	40.93 (12.5)	42.54 (18.8)	
Change	−2.18 (8.8)	−4.02 (7.4)	0.10 (10.1)	
Change difference	−4.12 (−9.24, 1.0)			0.113
**Stroke volume**				
Baseline	62.50 (19.0)	62.57 (20.8)	62.42 (17.2)	
Months 6	66.43 (22.8)	65.43 (26.5)	67.59 (18.3)	
Change	1.99 (17.3)	1.51 (21.5)	2.52 (11.5)	
Change difference	−1.01 (−12.93, 10.90)			0.864
**Left ventricle mass**				
Baseline	122.49 (30.5)	119.95 (26.0)	125.29 (35.3)	
Months 6	107.92 (27.4)	104.61 (27.0)	111.87 (27.9)	
Change	−14.29 (18.8)	−15.12 (17.6)	−13.42 (20.4)	
Change difference	−1.69 (−13.41, 10.02)			0.771
**Infarct mass**				
Baseline	24.48 (14.7)	21.32 (13.9)	28.40 (15.1)	
Months 6	19.10 (13.2)	17.00 (12.9)	21.70 (13.4)	
Change	−5.39 (11.0)	−4.33 (10.4)	−6.71 (11.8)	
Change difference	2.38 (−4.13, 8.90)			0.465
**Infarct mass (% LV mass)**				
Baseline	20.31 (12.23)	17.78 (11.0)	23.26 (13.1)	
Months 6	17.73 (12.0)	16.67 (12.1)	19.03 (12.1)	
Change	−2.19 (8.4)	−0.93 (8.3)	−3.75 (8.6)	
Change difference	2.73 (−2.27, 7.73)			0.276
**Infarct size downstream to the infarct-related artery**				
Baseline	19.92 (11.8)	17.60 (11.1)	22.78 (12.3)	
Months 6	17.73 (12.0)	16.67 (12.1)	19.03 (12.1)	
Change	−2.19 (8.4)	−0.93 (8.3)	−3.75 (8.6)	
Change difference	2.83 (−2.14, 7.79)			0.258
**Segments with microvascular obstruction**				
Baseline	1.00 (0.00, 3.00)	1.50 (0.00, 3.00)	0.00 (0.00, 2.00)	
Months 6	0.00 (0.00, 0.00)	0.00 (0.00, 0.00)	0.00 (0.00, 0.00)	
Change	−1.00 (−2.25, 0.00)	−2.00 (−3.00, 0.00)	0.00 (−2.00, 0.00)	
Change difference	−0.56 (−1.51, 0.39)			0.241

Data are shown as mean (SD). SD, standard deviation; CMR, cardiac magnetic resonances; IQR, interquartile range.

#### 3.3.3. Safety

Overall, 13 patients experienced adverse events, including eight serious adverse events and three events deemed possibly related to the study medication by the independent clinical events committee, with no significant differences between groups (Supplementary Table S8). No deaths were reported. The adverse events deemed possibly or likely related to the study medication included two cases of toxicodermia in the dapagliflozin group and one case of acute liver failure in the placebo group (Supplementary Table S9).

#### 3.3.4. Sensitivity Analyses

Repeat analysis in the per-protocol population was generally concordant with the main analysis (Supplementary Tables S10–S12). However, dapagliflozin was associated with a significantly higher reduction in NT-proBNP at 6-month (−11,125.33 pg/mL [767.7] vs. −793.23 pg/mL [585.5]; difference: −310.63 [−589.50, −310.70]; *p* = 0.030). Analysis after adjustment for T2DM status and MI detected a higher reduction in indexed LVESV in the dapagliflozin than placebo group, resulting in a significant difference at 6 months (adjusted difference: −4.92 [−9.8, −0.1]; *p* = 0.047; Supplementary Table S13). No other significant differences with the main analysis were detected.

## 4. Discussion

The key findings from our trial can be summarized as follows: (1) dapagliflozin was associated with a numerically higher reduction in ECV change at 6 months compared to placebo; (2) dapagliflozin led to numerically higher reduction in fibrosis and heart failure biomarkers than placebo at 6 months, with a significant reduction in NT-proBNP in the per-protocol setting; (3) dapagliflozin significantly reduced LVESV at 6 months compared to placebo.

Despite current advancement in STEMI management, including reduced time from symptoms to balloon, advancement in stent technology, and novel drugs for secondary prevention, STEMI patients still face a high risk of long-term adverse events, including all-cause mortality and hospitalization for heart failure [12,13], being even higher among patients with more advanced myocardial fibrosis [14,15]. In vitro and in vivo studies have shown that SGLT2is are able to prevent the development of myocardial fibrosis [6], leading to lower LV mass and ECV [7,16]. However, these studies focused either on patients with heart failure with reduced ejection fraction or patients with diabetes mellitus and stable CAD, while evidence of efficacy for preventing myocardial fibrosis among STEMI patients are still lacking. In this context, our study provides evidence of reduced myocardial fibrosis with administration of dapagliflozin even in patients with STEMI undergoing pPCI.

Specifically, dapagliflozin showed a numeric reduction in the development of cardiac fibrosis, with a 1.82 absolute reduction in ECV and higher reduction in P3NP compared to placebo in our study. In the Effects of Empagliflozin on Cardiac Structure in Patients With Type 2 Diabetes (EMPA-HEART) study, empagliflozin was associated with a 1.40 reduction in the percentage of ECV at 6 months compared to placebo among 74 patients with diabetes mellitus and CAD, supporting our results [7].

Several differences may explain the absence of significant differences between groups in our study compared to the EMPA-HEART trial. On one hand, STEMI patients may have a different tendency to develop myocardial fibrosis compared to patients with T2DM and stable CAD, as T2DM is associated with development of myocardial fibrosis and increased ECV [17], reflected in the higher baseline ECV among patients enrolled in the EMPA-HEART trial compared to the DAPA-STEMI trial [7]. In fact, our population was a relatively low-risk selected population, with a low presence of standard risk factors for CAD, including only 11.5% of patients having T2DM, and only 36% of patients enrolled suffering from hypertension, being the most common risk factor in our population, suggesting that SGLT2is may be more effective in reducing ECV in patients at higher risk. Notwithstanding these differences, the 1.82 reduction is in line with our prespecified treatment effect size, although the measure of variance was more than double than expected, suggesting that the low number of patients enrolled may have influenced our results [8].

In line with the hypothesis of dapagliflozin reducing myocardial fibrosis, we also found a 198 pg/mL reduction in 6-month PIIINP with dapagliflozin compared with placebo. PIIINP is a procollagen fragment released in the bloodstream from cardiac fibroblast in the presence of active collagen deposition, and circulating levels of PIIINP have been associated with myocardial fibrosis and increased mortality among heart failure patients [18], and drugs improving long-term outcomes in this population have been linked also to reduced PIIINP [19]. The hypothesis of a class-effect of SGLT2is in reducing circulating biomarkers of cardiac fibrosis is also supported by results of the EMPagliflozin outcomE tRial in Patients With chrOnic heaRt Failure (EMPEROR) studies, in which empagliflozin was associated with a 7% reduction in circulating PIIINP among patients with heart failure [20]. At difference with PIIINP, reduction in other biomarkers was generally of lower magnitude in our study. However, while PIIINP is a specific marker of myocardial fibrosis, these additional biomarkers are markers of general fibrosis occurring in different tissues, rather than specific indexes of myocardial fibrosis, and the lack of significant differences is in line with previous studies of SGLT2is, suggesting that the anti-fibrotic effects may be myocardium-specific. However, given the low sample size and the design of our study, assessing imaging-related signs of myocardial fibrosis rather than biochemical-related signs, such inference should be considered hypothesis generating.

Finally, our study also provides some mechanistic insights on the effects of dapagliflozin on heart failure. Specifically, dapagliflozin was associated with higher reduction in LVESV, and also reduced NT-pro-BNP in the per-protocol set. From a biological point of view, myocardial remodeling occurs at several levels, including anatomical, metabolic, and neurohormonal, and SGLT2is appear to interact with all three components of adverse remodeling [21]. Specifically, SGLT2is appear to shift the metabolism of the failing myocardium from a glucose-based to the normal free fatty acid-based metabolism, improving myocardial efficiency, while reducing infarct-related compensatory hypertrophy and development of spherical myocardial shapes, ultimately leading to lower LVESV and myocardial mass [5,21,22].

Regarding drug safety, dapagliflozin appeared to be well tolerated in our population, with only two patients experiencing serious adverse events possibly linked to the experimental study medication, being represented by skin eruptions. Although previous report indicated a possible association between the drug and skin eruptions as a cell-mediated delayed type of drug reaction [23], these appeared to be rare and without significant differences with placebo in our study. Interestingly, no ischemic events were recorded in the dapagliflozin group, as well as no urinary tract infections, highlighting the overall safety of the medication in the short-term.

## 5. Limitations

The results of our study should be evaluated in light of several limitations. First, the premature termination of the trial related to slow recruitment significantly impaired our ability to explore the relationship between dapagliflozin and ECV. Indeed, the wide adoption of SGLT2is in clinical practice, as well as the COVID-19 pandemics, significantly challenged patients’ recruitment [24], leading to the early discontinuation of the trial after enrolling less than half of the prespecified sample size, resulting in overall lack of statistical power. Second, our study mostly enrolled a relatively low-risk STEMI population, being hemodynamically stable, predominantly male with European ancestry, and with a relatively low prevalence of diabetes and standard risk factors for CAD, potentially limiting the generalizability of our results. Third, owing to the nature of study, the follow-up was brief, limiting the detection of possible long-term adverse events.

## 6. Conclusions

Among patients with STEMI undergoing pPCI, dapagliflozin did not reduce 6-month CMR-derived ECV compared to placebo. Given the premature termination of the study with enrollment of only 52 patients, these results should be considered hypothesis generating, and larger adequately powered studies are needed to derive final conclusions on dapagliflozin and myocardial fibrosis in STEMI patients undergoing pPCI.

## Figures and Tables

**Table 1:** Baseline characteristics of the enrolled population.

	Overall N = 52	Dapagliflozin N = 28	Placebo N = 24	*p*-Value
**Demographic data**				
Age (year), mean (SD)	59.7 (8.8)	59.4 (10.0)	60.1 (7.4)	0.774
Male sex, N (%)	49 (94.2%)	26 (92.9%)	23 (95.8%)	0.999
Current smokers, N (%)	23 (44.2%)	9 (32.1%)	14 (58.3%)	0.165
Body mass index (kg/m^2^), median (IQR)	27.18 (24.6, 30.1)	26.51 (24.6, 30.4)	28.32 (24.4, 29.4)	0.485
**Medical history**				
Type 2 diabetes mellitus, N (%)	5 (9.6%)	2 (7.1%)	3 (12.5%)	0.999
Hypertension, N (%)	19 (36.5%)	7 (25.0%)	12 (50.0%)	0.062
Hypercholesterolemia, N (%)	15 (28.8%)	9 (32.1%)	6 (25.0%)	0.571
Chronic kidney disease, N (%)	7 (13.5%)	3 (10.7%)	4 (16.7%)	0.690
Atrial fibrillation or flutter, N (%)	1 (1.9%)	0 (0.0%)	1 (4.2%)	0.462
Family history of premature, N (%) Coronary artery disease, N (%)	4 (7.7%)	3 (10.7%)	1 (4.2%)	0.615
Previous Stroke/transient, N (%) Ischemic attack, N (%)	0 (0.0%)	0 (0.0%)	0 (0.0%)	-
Previous PCI, N (%)	0 (0.0%)	0 (0.0%)	0 (0.0%)	-
Previous PVD, N (%)	1 (1.9%)	0 (0.0%)	1 (4.2%)	0.462
Previous Heart Failure, N (%)	0 (0.0%)	0 (0.0%)	0 (0.0%)	-
Previous Major bleeding, N (%)	0 (0.0%)	0 (0.0%)	0 (0.0%)	-
Previous history of COPD, N (%)	0 (0.0%)	0 (0.0%)	0 (0.0%)	-
**Medical therapy before admission**				
Aspirin, N (%)	1 (1.9%)	0 (0.0%)	1 (4.2%)	0.462
P2Y_12_ inhibitor, N (%)	0 (0.0%)	0 (0.0%)	0 (0.0%)	-
ACE inhibitor, N (%)	8 (15.4%)	3 (10.7%)	5 (20.8%)	0.313
ARBs, N (%)	3 (5.8%)	1 (3.6%)	2 (8.3%)	0.590
ARNI, N (%)	0 (0.0%)	0 (0.0%)	0 (0.0%)	-
Statins, N (%)	7 (13.5%)	3 (10.7%)	4 (16.7%)	0.690
Beta Blocker, N (%)	2 (3.8%)	0 (0.0%)	2 (8.3%)	0.208
Calcium channel blocker, N (%)	4 (7.7%)	1 (3.6%)	3 (12.5%)	0.324
Diuretics, N (%)	5 (9.6%)	1 (3.6%)	4 (16.7%)	0.169
MRAs, N (%)	0 (0.0%)	0 (0.0%)	0 (0.0%)	-
Proton-pump inhibitors, N (%)	3 (5.8%)	3 (10.7%)	0 (0.0%)	0.240
Anticoagulant treatment, N (%)	0 (0.0%)	0 (0.0%)	0 (0.0%)	-
**Laboratory measures**				
HbA1C (%), mean (SD)	5.86 (0.92)	5.75 (0.89)	6.01 (0.95)	0.149
Creatinine (mg/dL), mean (SD)	1.01 (0.33)	1.02 (0.40)	1.01 (0.23)	0.429
LDL cholesterol (mg/dL), mean (SD)	134.40 (38.0)	129.27 (39.6)	140.45 (35.9)	0.314
HDL cholesterol (mg/dL), mean (SD)	42.27 (10.1)	42.34 (10.7)	42.18 (9.6)	0.957

Abbreviations: ARBs, angiotensin II receptor blockers; ARNI, angiotensin receptor neprilysin inhibitor; COPD, chronic obstructive pulmonary disease; IQR, interquartile range; MRAs, mineralocorticoid receptor antagonists; PCI, percutaneous coronary intervention; PVD, peripheral vascular disease; SD, standard deviation.

## Data Availability

All data are available upon reasonable request to the corresponding author.

## Supplementary Materials

The following supporting information can be downloaded at: https://www.mdpi.com/article/10.3390/jcm15114061/s1, DAPA-STEMI trial organization. World Health Organization Trial Registration Details. DAPA-STEMI inclusion and exclusion criteria. Cardiac magnetic resonance acquisition protocol. Biomarker collection protocol. Supplementary Table S1. Procedural characteristics. Supplementary Table S2. Discharge data. Supplementary Table S3. Diabetes medications at admission and discharge. Supplementary Table S4. Baseline CMR characteristics. Supplementary Table S5. Change of fibrosis biomarkers levels from baseline to 6-month. Supplementary Table S6. Change of anthropometric variables from baseline to 6-month. Supplementary Table S7. Change of CMR variables from baseline to 6-month. Supplementary Table S8. Adverse events recorded during the study period. Supplementary Table S9. List of adverse events. Supplementary Table S10. Baseline characteristics in the per-protocol population. Supplementary Table S11. Changes in cardiac magnetic resonance parameters from baseline to 6-months in the per-protocol population. Supplementary Table S12. Changes of cardiac fibrosis markers in the per-protocol population. Supplementary Table S13. Sensitivity analysis after adjustment for T2DM. Supplementary Figure S1. Subgroup analyses.

## Abbreviations

ACE	Angiotensin-converting enzyme
ARB	Angiotensin receptor blocker
CAD	Coronary artery disease
CMR	Cardiac magnetic resonance
ECV	Extracellular volume
eGFR	Estimated glomerular filtration rate
Gal-3	Galectin-3
hs-TnI	High-sensitivity troponin I
LVEDV	Left ventricular end-diastolic volume
LVEF	Left ventricular ejection fraction
LVESV	Left ventricular end-systolic volume
LVM	Left ventricular mass
MI	Myocardial infarction
NT-proBNP	N-terminal pro–B-type natriuretic peptide
PICP	Pro-peptide of type I procollagen
PIIINP	Procollagen type III N-terminal peptide
pPCI	Primary percutaneous coronary intervention
SGLT2i	Sodium–glucose cotransporter 2 inhibitor
ST2	Suppression of tumorigenicity 2
STEMI	ST-segment elevation myocardial infarction
SV	Stroke volume
T2DM	Type 2 diabetes mellitus
