# Supplementary material for: Effect of Dapagliflozin on Myocardial Fibrosis After STEMI: A Double-Blind, Placebo-Controlled Randomized Trial
Source: J Clin Med. 2026 May 24;15(11):4061. doi: 10.3390/jcm15114061 (PMC13257374; doi:10.3390/jcm15114061)
Supplement: Supplementary file 1 [file jcm-15-04061-s001.zip › jcm-4290729-supplementary.pdf]

Supplementary Materials

Effect of Dapagliflozin on Myocardial Fibrosis After STEMI: A Double-Blind, Placebo-Controlled Randomized Trial

Luis Ortega-Paz, Claudio Laudani, Carlos Igor Morr, Alessandro Sionis, Pablo Vidal-Cales, Victor Arevalos, Rut Andrea, Oriol De Diego, Emilio Ortega, Francisco-Rafael Jimenez-Trinidad, Ana Paula Dantas, Dominick J. Angiolillo, Manel Sabaté, Jose T. Ortiz-Pérez and Salvatore Brugaletta

TABLE OF CONTENTS

DAPA-STEMI trial organization. 1

World Health Organization Trial Registration Details 2

DAPA-STEMI inclusion and exclusion criteria. 3

Cardiac magnetic resonance acquisition protocol. 5

Biomarker collection protocol. 8

SUPPLEMENTARY TABLES 9

Supplementary Table S1. Procedural characteristics. 9

Supplementary Table S2. Discharge data. 11

Supplementary Table S3. Diabetes medications at admission and discharge. 12

Supplementary Table S4. Baseline CMR characteristics. 13

Supplementary Table S5. Change of fibrosis biomarkers levels from baseline to 6-month. 14

Supplementary Table S6. Change of anthropometric variables from baseline to 6-month. 15

Supplementary Table S7. Change of CMR variables from baseline to 6-month. 16

Supplementary Table S8. Adverse events recorded during the study period. 18

Supplementary Table S9. List of adverse events. 19

Supplementary Table S10. Baseline characteristics in the per-protocol population. 21

Supplementary Table S11. Changes in cardiac magnetic resonance parameters from baseline to 6-months in the per-protocol population. 22

Supplementary Table S12. Changes of cardiac fibrosis markers in the per-protocol population. 24

Supplementary Table S13. Sensitivity analysis after adjustment for T2DM and myocardial infarction location. 25

SUPPLEMENTARY FIGURES 26

Supplementary Figure S1. Subgroup analyses. 26

DAPA-STEMI trial organization.

Committee	List of members
**Steering Committee**	Dr. Luis Ortega-Paz (Principal investigator)<br>Dr. Alessandro Sionis (site principal investigator)<br>Dr. Salvatore Brugaletta (Study chair)
**Data Safety Monitoring Board**	Dr. Andrea Ruperti<br>Dr. Riccardo Rinaldi
**Clinical Event Committee**	Dr. Manel Sabaté (Chair)<br>Dr. Víctor Arévalos<br>Dr. Pablo Vidal
**CRO**	Adelphy, Barcelona, Spain
**Monitoring**	Cristina Calle<br>Montserrat Perez
**Statistics**	Effice, Madrid, Spain<br>José Montes
**Core laboratory**	
**Cardiac magnetic resonance**	IDIBAPS Cardiac imaging group, Barcelona, Spain<br>Dr. José Tomás Ortiz-Pérez<br>Dr. Carlos Igor Morr
**Biomarkers**	IDIBAPS, Barcelona, Spain<br>Dr. Ana Paula Dantas

World Health Organization Trial Registration Details

- Trial Registration Number: NCT06619600
- Primary Registry and Trial Identifying Number: EudraCT, 2018-003105-25; ClinicalTrials.gov, NCT06619600
- Date of Registration in Primary Registry: September 14, 2018
- Secondary Identifying Numbers: Institutional Review Board protocol number HCB/2018/0960
- Source(s) of Monetary or Material Support: AstraZeneca (unrestricted grant ESR-19-14489)
- Primary Sponsor: Spanish Society of Cardiology, Ntra. Sra de Guadalupe, 5-7 28028 Madrid, Spain; Tel +34 91 724 23 70
- Contact for Public Queries: Luis Ortega-Paz, Email: Luis.Ortega@jax.ufl.edu
- Contact for Scientific Queries: Luis Ortega-Paz, MD, PhD, Division of Cardiology, University of Florida College of Medicine, Jacksonville, FL, USA; Tel: +1-904-244-3378
- Public Title: Effect of Dapagliflozin in Myocardial Fibrosis and Ventricular Function in STEMI Patients
- Scientific Title: Effect of Dapagliflozin on Myocardial Fibrosis and Ventricular Function in Patients with a ST-Segment Elevation Myocardial Infarction, DAPA-STEMI Trial
- Countries of Recruitment: Spain
- Health Condition(s) or Problem(s) Studied: Myocardial fibrosis and ventricular dysfunction in STEMI patients
- Intervention(s): Dapagliflozin 10 mg daily versus placebo for six months
- Key Inclusion Criteria: Patients aged 30-85 years with STEMI undergoing primary PCI, LVEF ≤50%, hemodynamically stable (Killip Class I), and no contraindication to CMR
- Key Exclusion Criteria: Pregnant or lactating women, type 1 diabetes, prior SGLT2i use, severe liver or kidney disease, malignancy, or contraindications to CMR
- Study Type: Phase III, multicenter, randomized, double-blind, placebo-controlled trial
- Date of First Enrollment: May 27, 2021
- Target Sample Size: 94 patients (53 randomized due to early termination)
- Recruitment Status: Recruitment closed; trial completed
- Primary Outcome(s): Change in extracellular volume (ECV) of the remote myocardium from baseline to six months, measured by CMR
- Key Secondary Outcomes: Changes in circulating biomarkers (e.g., PICP, PIIINP, Galectin-3) and additional CMR parameters (e.g., LV mass, EF)

DAPA-STEMI inclusion and exclusion criteria.

Inclusion criteria:

1. Patients between 30–85 years of age.
2. Patients with first infarction with ST-segment elevation documented in an ambulance or a cardiac catheterization laboratory (ST-segment elevation ≥2 mm in at least two contiguous leads) less than <12 hours after onset of symptoms that last ≥ 20 min, that is treated with primary percutaneous cardiac intervention. The target lesion must be a de novo lesion located in a native vessel.
3. The patient understands and accepts clinical monitoring and CMR.
4. The patient must be hemodynamically stable (Killip classification 1) at the time of the initial CMR.
5. A left ventricular ejection fraction ≤50% at baseline echocardiogram.

Exclusion criteria:

1. Pregnant or lactating women.
2. Type 1 diabetes mellitus.
3. Previous treatment with SGLT2i.
4. Severe liver disease (Child-Pugh C).
5. Kidney disease defined as stage III or worse (eGFR <45 mL/min).
6. Systolic blood pressure <90 mmHg at the screening visit.
7. Malignancy (receiving active treatment) or other life-threatening diseases.

8. Any contraindication to CMR (e.g., claustrophobia, cerebrovascular implants, metal implants, penetrating eye injury, or exposure to metal fragments in the eye that require medical attention, hemodynamic or electrical instability).
9. Previous complicated urinary tract infection in men or repeated urinary infection in women.
10. Patients treated with fibrinolytic therapy.

Cardiac magnetic resonance acquisition protocol.

CMR was performed at baseline and 6 months following randomization on a 3T scanner (ARCHITECT, General Electric, Wisconsin, USA) equipped with a phased-array body surface coil and a cardiac-dedicated package with electrocardiogram gating. The following images were obtained within the study: conventional steady-state free-precession cine images covering the entire left ventricle in the short axis, with a slice thickness of 8 mm and no gap between slices, to calculate indexed end-diastolic volume, indexed end-systolic volume, LVEF, and LV mass. T2 maps were obtained prior to contrast injection in the short axis. T1 maps were acquired before gadobutrol administration (0.2 mmol/kg, Gadovist®, Bayer Hispania, Barcelona) and 15 minutes later using a modified Look-Locker inversion-recovery sequence (MOLLI) with an embedded motion correction algorithm in the short axis orientation at the basal, mid-ventricular, and apical levels, as well as in the four- and two-chamber views. Pre-contrast T1 maps were obtained using a 5-(3)-3 scheme, and post-contrast T1 maps were obtained with a 4(1)3(1)2 scheme. Late enhancement images were acquired 10 minutes after gadolinium administration using a standard ECG-gated T1-weighted segmented inversion-recovery gradient-echo sequence during repeated breath-holds in the same short axis locations and long axis views as the cine images.

All scans were analyzed at a core laboratory facility (IDIBAPS Cardiac Imaging Group, Barcelona, Spain) by experienced cardiologists (CIM and JTO) in CMR postprocessing to generate a study clinical report. The scans were then pseudo-anonymized and analyzed by a single operator in accordance with the guidelines from the Society for Cardiovascular Magnetic Resonance and the European Society of Cardiovascular Imaging for reporting CMR examinations.[11] The baseline and 24-week scans were analyzed in pairs and side-by-side to minimize intra-observer variability, with the operator blinded to treatment allocation.

Native T1 and ECV values derivation

Whenever possible, three regions of interest (ROIs) of at least 1 cm² were drawn in three distinct LV myocardial segments in the remote non-infarcted myocardium among the basal and midventricular slices, avoiding areas with image artifacts. Special attention was given to appropriate co-registration of the tracings between the baseline and follow-up studies to minimize interstudy variability and misregistration. The corresponding late-gadolinium enhancement short-axis images to the T1 maps were checked to avoid placing ROIs in areas with acute or chronic myocardial infarction. The mean value of all ROIs were labeled as the remote native T1 values for the baseline and follow-up studies. The mean myocardial ECV for both baseline and follow-up studies were computed using the following formula:

$$ECV = (1 - \text{hematocrit}) \times \left[\frac{1}{\text{postcontrast } T1_{my}} - \frac{1}{\text{native } T1_{my}}\right] \div \left[\frac{1}{\text{postcontrast } T1_{bp}} - \frac{1}{\text{native } T1_{bp}}\right]$$

where postcontrast $T1_{my}$ is postcontrast myocardial T1; native$T1_{my}$ is native myocardial T1; postcontrast $T1_{bp}$ is postcontrast blood pool T1 and native $T1_{bp}$ is native blood pool T1.

LV function, volumes and infarct size calculations

Circle CVI 42 Medical Image post-processing software for Cardiac Magnetic Resonance, Version 5.12.1 (Alberta, Canada), was used for these calculations. Briefly, an artificial intelligence-based algorithm automatically delineates the endocardial and epicardial contours at end-systole and end-diastole. Manual corrections were performed, if necessary, to compute the LV end-systolic and end-diastolic volumes and LVEF.

The infarct zone was quantified semi-automatically. Initially, the region with the highest signal intensity in the infarct core was identified, and the infarcted tissue was defined as the area

with signal intensity above 50% of the maximum pixel intensity (full width at half maximum intensity method). Regions of microvascular obstruction were included in the quantification. Finally, the infarcted areas were summed and divided by the total mass of the left ventricular wall to calculate the infarct size as a percentage of the total LV mass.

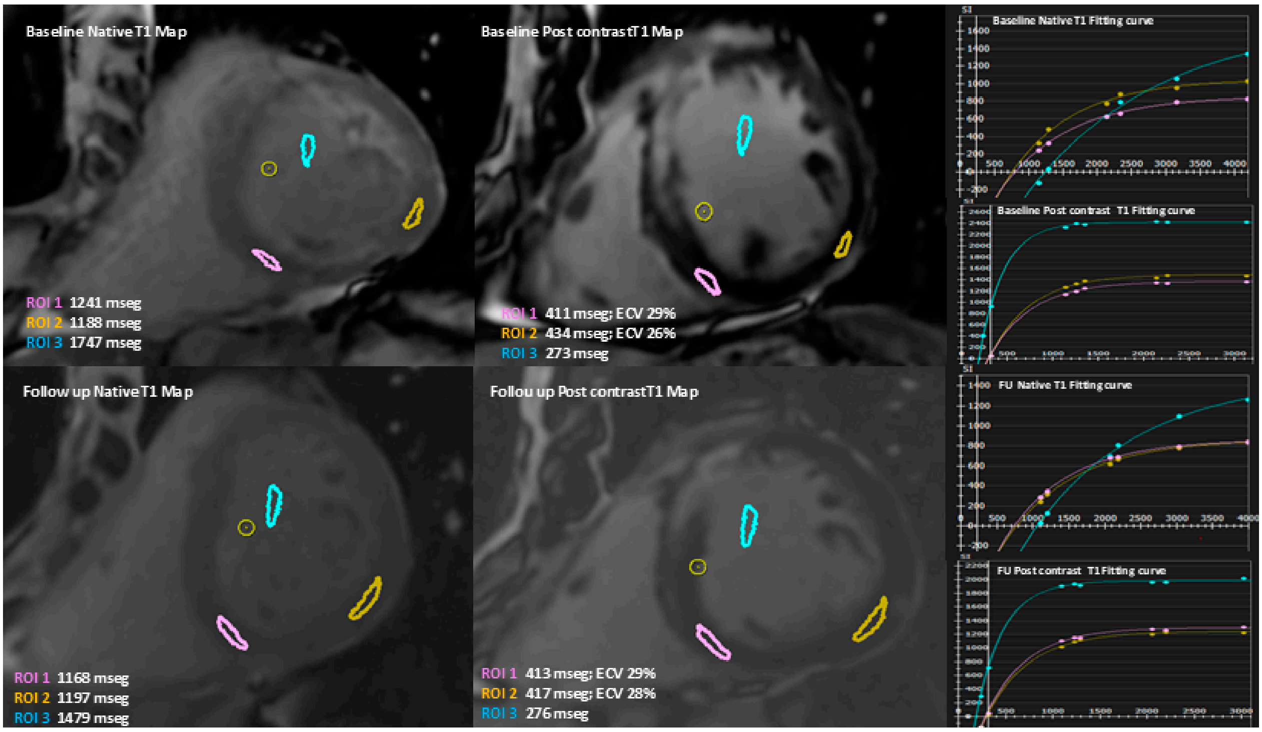

Anteroseptal myocardial infarction. Baseline and 6-month follow-up CMR. Quantification of T1 mapping and extracellular volume (ECV). ROIs were delineated in remote myocardial regions on pre- and post-contrast images, and ECV was calculated using hematocrit values.

Biomarker collection protocol.

Venous blood and urine samples for biomarker analysis were collected at 3 time points: baseline (i.e., after consent and prior to randomization) and at three and six months following randomization. Blood samples were collected in serum separation BD Vacutainer® SST™ Tubes, immediately chilled, and then centrifuged as soon as possible at 2500 g at 4°C for 10 minutes. The samples were aliquoted and stored at -80°C at the laboratory of Experimental Cardiology (IDIBAPS, Barcelona, Spain) until batch analysis. Upon completion of the trial, samples were analyzed for the concentration of C-terminal propeptide of type I procollagen, N-terminal propeptide of type III procollagen, Galectin-3, and Suppression of Tumorigenicity 2. The levels of C-terminal propeptide of type I procollagen and N-terminal propeptide of type III procollagen were determined by enzyme-linked immunoassay (ELISA) kit (Elabscience Biotechnology Co., Ltd). Galectin-3 and Suppression of Tumorigenicity 2 were determined by bead-based multiplex immunoassay (R&D Systems, Inc).

SUPPLEMENTARY TABLES

Supplementary Table S1. Procedural characteristics.

	Total N=52	DAPA N=28	Placebo N=24	p
**Clinical and EKG variables**				
Time from symptom onset to wire crossing (h), median (IQR)	3.1 (2.1-4.9)	3.0 (2.1,6.2)	3.5 (2.2,4.3)	0.970
Myocardial infarction localization				0.754
Anterior	39 (75.0%)	20 (71.4%)	19 (79.2%)	
Lateral	2 (3.8%)	1 (3.6%)	1 (4.2%)	
Inferior	11 (21.2%)	7 (25.0%)	4 (16.7%)	
Killip class				0.529
I	45 (86.5%)	24 (85.7%)	21 (87.5%)	
II	6 (11.5%)	4 (14.3%)	2 (8.3%)	
III	0 (0.0%)	0 (0.0%)	0 (0.0%)	
IV	1 (1.9%)	0 (0.0%)	1 (4.2%)	
**PCI variables**				
Target segment				0.928
Proximal RCA	5 (9.6%)	3 (10.7%)	2 (8.3%)	
Mid RCA	3 (5.8%)	1 (3.6%)	2 (8.3%)	
Prox LAD	23 (44.2%)	12 (42.9%)	11 (45.8%)	
Mid LAD	15 (28.8%)	9 (32.1%)	6 (25.0%)	
1st DIAG	1 (1.9%)	0 (0.0%)	1 (4.2%)	
Prox LCX	1 (1.9%)	0 (0.0%)	1 (4.2%)	
Mid LCX	2 (3.8%)	1 (3.6%)	1 (4.2%)	
2nd OB Marg	1 (1.9%)	1 (3.6%)	0 (0.0%)	
3rd OB Marg	1 (1.9%)	1 (3.6%)	0 (0.0%)	
Number of diseased vessels, mean (SD)	1.48 (0.67)	1.54 (0.69)	1.42 (0.65)	0.772
1 vessel	32 (61.5%)	16 (57.1%)	16 (66.7%)	
2 vessels	15 (28.8%)	9 (32.1%)	6 (25.0%)	
3 vessels	5 (9.6%)	3 (10.7%)	2 (8.3%)	
Pre-procedural TIMI flow				0.541
0	40 (76.9%)	22 (78.6%)	18 (75.0%)	
1	2 (3.8%)	0 (0.0%)	2 (8.3%)	
2	4 (7.7%)	2 (7.1%)	2 (8.3%)	
3	6 (11.5%)	4 (14.3%)	2 (8.3%)	
Patients receiving drug-eluting stent implantation	49 (94.2%)	27 (96.4%)	22 (91.7%)	0.590
Number of implanted stents, mean (SD)	1.31 (0.65)	1.30 (0.61)	1.32 (0.72)	0.999
1	38 (77.6%)	21 (77.8%)	17 (77.3%)	
2	8 (16.3%)	4 (14.8%)	4 (18.2%)	
3	2 (4.1%)	2 (7.4%)	0 (0.0%)	
4	1 (2.0%)	0 (0.0%)	1 (4.5%)	
Post-PCI TIMI flow				0.092

0	3 (5.8%)	0 (0.0%)	3 (14.3%)	
2	0 (0.0%)	0 (0.0%)	0 (0.0%)	
3	49 (94.2%)	28 (100.0%)	21 (83.3%)	
Post-PCI killip class				0.726
1	49 (94.2%)	27 (96.4%)	22 (91.7%)	
2	2 (3.8%)	1 (3.6%)	1 (4.2%)	
4	1 (1.9%)	0 (0.0%)	1 (4.2%)	

Supplementary Table S2. Discharge data.

	Total N=52	DAPA N=28	Placebo N=24	p
**Discharge data**				
Time in hospital (days), median (IQR)	4.5 (3.1,6.4)	4.5 (3.3,6.4)	4.2 (2.6,6.3)	0.603
**Treatment at discharge**				
Aspirin	52 (100.0%)	28 (100.0%)	24 (100.0%)	0.999
P2Y12 inhibitor	52 (100.0%)	28 (100.0%)	24 (100.0%)	0.999
P2Y12 inhibitor type				0.589
Clopidogrel	13 (25.0%)	8 (28.6%)	5 (20.8%)	
Prasugrel	27 (51.9%)	15 (53.6%)	12 (50.0%)	
Ticagrelor	12 (23.1%)	5 (17.9%)	7 (29.2%)	
ACE inhibitor	30 (57.7%)	17 (60.7%)	13 (54.2%)	0.634
ACE inhibitor type				0.132
Lisinopril	1 (3.3%)	1 (5.9%)	0 (0.0%)	
Enalapril	18 (60.0%)	12 (70.6%)	6 (46.2%)	
Ramipril	11 (36.7%)	4 (23.5%)	7 (53.8%)	
ARBs	11 (21.2%)	6 (21.4%)	5 (20.8%)	0.958
ARBs type				0.999
Losartan	4 (36.4%)	2 (33.3%)	2 (40.0%)	
Valsartan	7 (63.6%)	4 (66.7%)	3 (60.0%)	
ARNI	2 (3.8%)	1 (3.6%)	1 (4.2%)	0.999
Statins	52 (100.0%)	28 (100.0%)	24 (100.0%)	0.999
Beta Blocker (Bisoprolol)	41 (78.8%)	24 (85.7%)	17 (70.8%)	0.190
CCB (Amlodipine)	4 (7.7%)	2 (7.1%)	2 (8.3%)	0.999
Diuretics	5 (9.6%)	2 (7.1%)	3 (12.5%)	0.652
Diuretic class				0.999
Thiazide	2 (40.0%)	1 (50.0%)	1 (33.3%)	
Loop diuretics	3 (60.0%)	1 (50.0%)	2 (66.7%)	
MRAs	20 (38.5%)	13 (46.4%)	7 (29.2%)	0.202
Proton-pump inhibitors	52 (100.0%)	28 (100.0%)	24 (100.0%)	0.999
Anticoagulant treatment	11 (21.2%)	6 (21.4%)	5 (20.8%)	0.958
Anticoagulant type				0.999
Vitamin K antagonists	9 (81.8%)	5 (83.3%)	4 (80.0%)	
Directly acting oral	2 (18.2%)	1 (16.7%)	1 (20.0%)	

Supplementary Table S3. Diabetes medications at admission and discharge.

	Total<br>N=5	DAPA<br>N=2	Placebo<br>N=3	p-value
**Baseline medication**				
Metformin	4 (80.0%)	2 (100.0%)	2 (66.7%)	0.999
Sulfonylureas	1 (20.0%)	1 (50.0%)	0 (0.0%)	0.400
Meglitinides	1 (20.0%)	0 (0.0%)	1 (33.3%)	0.999
Thiazolidinediones	0 (0.0%)	0 (0.0%)	0 (0.0%)	-
Alpha-gluco inhibitors	0 (0.0%)	0 (0.0%)	0 (0.0%)	-
Dipeptidylpeptidase Inhibitors	1 (20.0%)	1 (50.0%)	0 (0.0%)	0.400
GLP-1	0 (0.0%)	0 (0.0%)	0 (0.0%)	-
Insulin	2 (40.0%)	1 (50.0%)	1 (33.3%)	0.999
**Discharge medication**				
Metformin	4 (80.0%)	2 (100.0%)	2 (66.7%)	0.999
Sulfonylureas	1 (20.0%)	0 (0.0%)	1 (33.3%)	0.999
Meglitinides	1 (20.0%)	0 (0.0%)	1 (33.3%)	0.999
Thiazolidinediones	0 (0.0%)	0 (0.0%)	0 (0.0%)	-
Alpha-gluco inhibitors	0 (0.0%)	0 (0.0%)	0 (0.0%)	-
Dipeptidylpeptidase Inhibitors	1 (20.0%)	1 (50.0%)	0 (0.0%)	0.400
GLP-1	0 (0.0%)	0 (0.0%)	0 (0.0%)	-
Insulin	2 (40.0%)	1 (50.0%)	1 (33.3%)	0.999

Supplementary Table S4. Baseline CMR characteristics.

	Total N=52	DAPA N=28	Placebo N=24	p
**Baseline cardiac magnetic resonance**, mean (SD)				
LV ejection fraction (%)	42.4 (5.0)	41.6 (5.4)	43.3 (4.3)	0.223
LV end-diastolic volume (mL)	149.3 (34.5)	148.9 (31.0)	149.7 (38.8)	0.941
LV end-systolic volume (mL)	85.1 (19.9)	86.5 (16.7)	83.4 (23.3)	0.588
Stroke volume (mL)	63.5 (19.3)	63.9 (21.2)	63.0 (17.6)	0.882
Left ventricle mass (grams)	123.18 (29.57)	120.79 (25.13)	125.78 (34.14)	0.565
Infarct mass Infarct related artery (grams)	24.95 (14.96)	21.61 (13.79)	28.84 (15.62)	0.083
Infarct size Infarct related artery (% of LV mass)	20.31 (12.23)	17.78 (11.02)	23.26 (13.13)	0.108

EKG, Electrocardiogram; PCI, Percutaneous Coronary Intervention; LV, Left ventricular; SD, standard deviation; IQR, interquartile range; MI, myocardial infraction

Supplementary Table S5. Change of fibrosis biomarkers levels from baseline to 6-month.

	Total<br>N=52	DAPA<br>N=28	Placebo<br>N=24	p
**PICP (pg/mL)**				
Baseline	1200.48 (317.9)	1130.19 (346.9)	1280.31 (270.8)	
Month 6	1061.73 (401.17)	1084.75 (404.0)	1029.65 (387.5)	
Change from baseline	-168.84 (469.76)	-111.74 (481.4)	-250.66 (452.1)	
Change difference		65.61 (-168.7,299.9)		0.576
**PIIINP (pg/mL)**				
Baseline	693.55 (354.0)	644.11 (321.6)	726.34 (375.6)	
Month 6	621.56 (365.0)	532.33 (330.2)	729.99 (359.3)	
Change from baseline	-95.23 (506.7)	-177.00 (416.1)	3.64 (553.8)	
Change difference		-198.17 (-401.0,4.7)		0.055
**Galectin-3 procollagen (ng/mL)**				
Baseline	1329.83 (894.2)	1127.85 (474.1)	1525.28 (1135.6)	
Month 6	1538.95 (1133.2)	1537.45 (1202.4)	1653.01 (1234.9)	
Change from baseline	191.36 (1505.4)	358.21 (1286.4)	127.74 (1797.6)	
Change difference		-159.30 (-887.2,568.6)		0.706
**sST-2 (ng/mL)**				
Baseline	1411.92 (657.0)	1418.31 (703.8)	1467.48 (626.1)	
Month 6	1344.30 (669.5)	1261.85 (736.4)	1442.58 (561.0)	
Change from baseline	-14.63 (773.3)	-42.07 (856.2)	-24.91 (684.5)	
Change difference		-138.61 (-516.4,239.1)		0.464
**Troponin I peak (ng/dL)**				
Baseline	166,697.16 (153,693.1)	133,827.0 (128005.7)	203358.8 (169650.0)	
Month 6	22.30 (36.5)	26.57 (47.0)	17.36 (13.4)	
Change from baseline	-161,998.01 (158,502.5)	-127,153 (113073.0)	-219529 (189545.4)	
Change difference		15.76 (-7.8,39.3)		0.363
**NT-proBNP peak (pg/mL)**				
Baseline	1501.10 (1075.1)	1260.13 (779.2)	1799.65 (1330.7)	
Month 6	448.14 (638.5)	271.12 (199.7)	622.61 (830.1)	
Change from baseline	-1070.47 (736.98)	-1102.81 (726.3)	-1133.47 (856.9)	
Change difference		-226.04 (-531.3,79.2)		0.142

Data are shown as mean (SD)
sst-2, Soluble suppression of tumorigenicity-2; PICP, C-terminal propeptide of type I procollagen; P3NP, N-terminal propeptide of type III; SD, standard deviation

Supplementary Table S6. Change of anthropometric variables from baseline to 6-month.

	N	Mean (SD)	95% CI	p-value
**Body weight (cm)**				
Dapagliflozin				
Baseline	28	78.96 (10.4)	(74.9,83.0)	
6-month	27	76.73 (9.5)	(73.0,80.5)	
Change	27	-1.86 (5.0)	(-3.8,0.1)	0.062
Placebo				
Baseline	24	81.79 (12.1)	(76.7,86.9)	
6-month	24	79.96 (12.4)	(74.7,85.2)	
Change	24	-1.83 (6.1)	(-4.4,0.7)	
Between-group change difference		0.30 (-3.03,3.62)		0.858
**Abdominal circumference (cm)**				
Dapagliflozin				
Baseline	28	99.30 (8.1)	(96.2,102.5)	
6-month	26	95.06 (7.3)	(92.1,98.0)	
Change	26	-3.92 (6.1)	(-6.4,-1.5)	**0.003**
Placebo				
Baseline	24	101.48 (13.1)	(95.9,107.0)	
6-month	24	101.17 (9.8)	(97.0,105.3)	
Change	24	-0.31 (7.5)	(-3.5,2.9)	0.840
Between-group change difference		-3.66 (-7.84,0.52)		0.085
**Hip circumference (cm)**				
Dapagliflozin				
Baseline	28	96.65 (9.4)	(92.6,100.7)	
6-month	26	96.02 (7.8)	(92.7,99.4)	
Change	26	-0.19 (6.7)	(-3.2,2.9)	0.199
Placebo				
Baseline	24	98.42 (6.9)	(95.5,101.3)	
6-month	24	97.98 (7.1)	(95.0,101.0)	
Change	24	-0.44 (5.2)	(-2.6,1.8)	0.901
Between-group change difference		0.91 (-2.84,4.65)		0.628

Supplementary Table S7. Change of CMR variables from baseline to 6-month.

	N	Mean (SD)	95% CI	p-value
**Left ventricular ejection fraction**				
Dapagliflozin				
Baseline	26	41.53 (5.6)	(39.3,43.8)	
6-month	26	47.64 (9.5)	(43.8,51.5)	
Change	26	6.11 (7.3)	(3.2,9.0)	**<0.001**
Placebo				
Baseline	21	43.49 (3.7)	(41.8,45.2)	
6-month	21	47.04 (9.5)	(42.7,51.4)	
Change	21	3.54 (7.5)	(0.1,7.0)	**0.042**
**End diastolic volume of the left ventricle**				
Dapagliflozin				
Baseline	26	147.19 (30.7)	(134.8,159.6)	
6-month	26	147.83 (36.0)	(133.3,162.4)	
Change	26	0.64 (20.4)	(-7.6,8.9)	0.875
Placebo				
Baseline	21	149.04 (40.8)	(130.5,167.6)	
6-month	21	149.39 (46.8)	(128.1,170.7)	
Change	21	0.34 (17.4)	(-7.6,8.3)	0.929
**End systolic volume of the left ventricle**				
Dapagliflozin				
Baseline	26	85.65 (16.8)	(78.9,92.5)	
6-month	26	77.27 (24.4)	(67.4,87.1)	
Change	26	-8.39 (13.6)	(-13.9,-2.9)	**0.004**
Placebo				
Baseline	21	82.69 (24.8)	(71.4,94.0)	
6-month	21	81.83 (36.6)	(65.2,98.5)	
Change	21	-0.86 (19.1)	(-9.6,7.8)	0.839
**Stroke volume**				
Dapagliflozin				
Baseline	20	62.57 (20.8)	(52.9,72.3)	
6-month	23	65.43 (26.5)	(54.0,76.9)	
Change	19	1.51 (21.5)	(-8.9,11.9)	0.764
Placebo				
Baseline	17	62.42 (17.2)	(53.6,71.3)	
6-month	20	67.59 (18.3)	(59.0,76.1)	
Change	17	2.52 (11.5)	(-3.4,8.5)	0.382
**Left ventricle mass**				
Dapagliflozin				
Baseline	23	119.95 (26.0)	(108.7,131.2)	
6-month	25	104.61 (27.0)	(93.4,115.8)	

Change	22	-15.12 (17.6)	(-22.9,-7.3)	**<0.001**
Placebo				
Baseline	21	125.29 (35.3)	(109.2,141.3)	
6-month	21	111.87 (27.9)	(99.2,124.6)	
Change	21	-13.42 (20.4)	(-22.7,-4.1)	**0.008**
**Infarct mass**				
Dapagliflozin				
Baseline	26	21.32 (13.9)	(15.7,26.9)	
6-month	26	17.00 (12.9)	(11.8,22.2)	
Change	26	-4.33 (10.4)	(-8.5,-0.1)	**0.008**
Placebo				
Baseline	21	28.40 (15.1)	(21.5,35.3)	
6-month	21	21.70 (13.4)	(15.6,27.8)	
Change	21	-6.71 (11.8)	(-12.1,-1.3)	**0.017**
**Infarct size downstream to the infarct related artery**				
Dapagliflozin				
Baseline	26	17.60 (11.1)	(13.1,22.1)	
6-month	26	16.67 (12.1)	(11.8,21.6)	
Change	26	-0.93 (8.3)	(-4.3,2.4)	0.188
Placebo				
Baseline	21	22.78 (12.3)	(17.2,28.4)	
6-month	21	19.03 (12.1)	(13.5,24.5)	
Change	21	-3.75 (8.6)	(-7.6,0.1)	0.058

Supplementary Table S8. Adverse events recorded during the study period.

	Total N=52	DAPA N=28	Placebo N=24	p-value
Adverse events	13 (25.0%)	4 (14.3%)	9 (37.5%)	0.106
Serious adverse event	8 (15.4%)	2 (7.1%)	6 (25.0%)	0.123
Possible-likely relation to study medication*	3 (5.8%)	2 (7.1%)	1 (4.2%)	0.646
Death	0 (0.0%)	0 (0.0%)	0 (0.0%)	-
Myocardial infarction	1 (1.9%)	0 (0.0%)	1 (4.2%)	0.416
Revascularization (without MI)	1 (1.9%)	0 (0.0%)	1 (4.2%)	0.416
Stroke	1 (1.9%)	0 (0.0%)	1 (4.2%)	0.416
Heart failure hospitalization	1 (1.9%)	0 (0.0%)	1 (4.2%)	0.416
Acute liver failure	2 (3.9%)	0 (0.0%)	2 (8.3%)	0.493
Other	8 (15.4%)	4 (14.3%)	4 (16.7%)	0.812

Data are shown as n (%)
ACS, Acute coronary syndrome
*DAPA group: Toxicodermia, Skin eruption; Placebo group: Acute liver failure.

Supplementary Table S9. List of adverse events.

Patient	Group	Start date	Description	SAE	Severity	Action	Relationship	Outcome	Stop date
**Adverse events**									
1	Placebo	22/03/2022	Acute pericarditis	No	Moderate	Yes	Unlikely	Resolved	29/03/2022
14	Placebo	23/11/2022	Acute coronary syndrome	Yes	Severe	Yes	Not related	Resolved	07/12/2022
16	Placebo	03/07/2023	Carotid endarterectomy surgery	Yes	Severe	No	Not related	Resolved	04/07/2023
24	Placebo	25/12/2023	Acute liver failure	Yes	Severe	Yes	Not related	Resolved	02/01/2024
277-1	Placebo	23/06/2021	Heart failure hospitalization	Yes	Severe	Yes	Unlikely	Resolved	07/07/2021
277-7	Dapagliflozin	16/12/2021	Syncope	No	Mild	Yes	Unlikely	Resolved	16/12/2021
277-10	Placebo	01/01/2022	Syncope	No	Mild	Yes	Unlikely	Resolved	01/01/2022
277-11	Placebo	04/09/2021	Ischemic stroke	No	Moderate	No	Not related	Resolved	04/09/2021
277-11	Placebo	23/02/2022	Intraventricular thrombosis	No	Moderate	Yes	Not related	Resolved	28/02/2022
277-12	Dapagliflozin	08/02/2022	Epigastric pain	No	Mild	Yes	Unlikely	Resolved	08/02/2022
277-15	Dapagliflozin	16/10/2021	Toxicoderma	Yes	Severe	Yes	Possibly	Resolved	20/10/2021
277-16	Dapagliflozin	25/10/2021	Rash on the chest	Yes	Mild	Yes	Likely	Resolved	06/11/2021
277-23	Placebo	21/02/2023	Target-vessel Revascularization without target-lesion revascularization	Yes	Moderate	Yes	Not related	Resolved	21/02/2023
277-24	Placebo	29/10/2022	Acute liver failure	Yes	Severe	Yes	Likely	Resolved	04/11/2022

Serious adverse events									
14	Placebo	23/11/2022	Acute coronary syndrome	Yes	Severe	Yes	Not related	Resolved	07/12/2022
16	Placebo	03/07/2023	Carotid endarterectomy surgery	Yes	Severe	No	Not related	Resolved	04/07/2023
24	Placebo	25/12/2023	Acute liver failure	Yes	Severe	Yes	Not related	Resolved	02/01/2024
277-1	Placebo	23/06/2021	Heart failure hospitalization	Yes	Severe	Yes	Unlikely	Resolved	07/07/2021
277-15	Dapagliflozin	16/10/2021	Toxicoderma	Yes	Severe	Yes	Possibly	Resolved	20/10/2021
277-16	Dapagliflozin	25/10/2021	Rash on the chest	Yes	Mild	Yes	Likely	Resolved	06/11/2021
277-23	Placebo	21/02/2023	Target-vessel Revascularization without target-lesion revascularization	Yes	Moderate	Yes	Not related	Resolved	21/02/2023
277-24	Placebo	29/10/2022	Acute liver failure	Yes	Severe	Yes	Likely	Resolved	04/11/2022

Supplementary Table S10. Baseline characteristics in the per-protocol population.

	Overall<br>N=39	Dapagliflozin<br>N=21	Placebo<br>N=18	p-value
**Demographic data**				
Age (year), mean (SD)	60.25 (9.12)	60.66 (10.43)	59.78 (7.59)	0.763
Male sex, N (%)	37 (94.87%)	20 (95.24%)	17 (94.44%)	0.999
Current smokers	17 (43.59%)	6 (28.57%)	11 (61.11%)	0.127
Body mass index (kg/m^2)	26.85 (3.67)	26.70 (3.16)	27.03 (4.28)	0.789
**Medical history**				
Type 2 diabetes mellitus	3 (7.69%)	2 (9.52%)	1 (5.56%)	0.999
Hypertension	13 (33.33%)	6 (28.57%)	7 (38.89%)	0.733
Hypercholesterolemia	11 (28.21%)	9 (42.86%)	2 (11.11%)	0.066
Chronic kidney disease	5 (12.82%)	3 (14.29%)	2 (11.11%)	0.999
Atrial fibrillation or flutter	1 (2.56%)	0 (0.00%)	1 (5.56%)	0.482
Family history of premature coronary artery disease	3 (7.69%)	2 (9.52%)	1 (5.56%)	0.999
Previous Stroke/transient ischemic attack	0 (0.0%)	0 (0.0%)	0 (0.0%)	-
Previous PCI	0 (0.0%)	0 (0.0%)	0 (0.0%)	-
Previous PVD	0 (0.0%)	0 (0.0%)	0 (0.0%)	-
Previous Heart Failure	0 (0.0%)	0 (0.0%)	0 (0.0%)	-
Previous Major bleeding	0 (0.0%)	0 (0.0%)	0 (0.0%)	-
Previous history of COPD	0 (0.0%)	0 (0.0%)	0 (0.0%)	-
**Medical therapy before admission**				
Aspirin	0 (0.0%)	0 (0.0%)	1 (4.2%)	-
$P2Y_{12}$ inhibitor	0 (0.0%)	0 (0.0%)	0 (0.0%)	-
ACE inhibitor	6 (15.4%)	3 (14.29%)	3 (14.29%)	0.999
ARBs	2 (5.13%)	1 (4.76%)	1 (5.56%)	0.999
ARNI	0 (0.0%)	0 (0.0%)	0 (0.0%)	-
Statins	4 (10.26%)	3 (14.3%)	1 (5.56%)	0.598
Beta Blocker	2 (3.8%)	0 (0.0%)	0 (0.0%)	-
Calcium channel blocker	2 (5.13%)	1 (4.76%)	1 (5.56%)	0.999
Diuretics	3 (7.69%)	1 (4.76%)	2 (11.11%)	0.577
MRAs	0 (0.0%)	0 (0.0%)	0 (0.0%)	-
Proton-pump inhibitors	3 (7.69%)	3 (14.3%)	0 (0.0%)	0.226
Anticoagulant treatment	0 (0.0%)	0 (0.0%)	0 (0.0%)	-
**Laboratory measures**				
HbA1C (%)	5.78 (0.81)	5.82 (1.02)	5.72 (0.43)	0.700
Creatinine (mg/dL)	1.01 (0.35)	1.06 (0.46)	0.95 (0.16)	0.357
LDL cholesterol (mg/dL)	139.86 (38.17)	140.41 (37.09)	140.41 (37.09)	0.936
HDL cholesterol (mg/dL)	42.64 (10.61)	43.17 (11.25)	42.06 (10.16)	0.758

Abbreviations: ARBs, Angiotensin II Receptor Blockers; ARNI, Angiotensin receptor neprilysin inhibitor; COPD, chronic obstructive pulmonary disease; IQR, interquartile range; MRAs, Mineralocorticoid receptor antagonists; PCI, Percutaneous coronary intervention; PVD, Peripheral Vascular Disease; SD, standard deviation.

Supplementary Table S11. Changes in cardiac magnetic resonance parameters from baseline to 6-months in the per-protocol population.

	Total N=39	DAPA N=21	Placebo N=18	p-value
**Time between CMRs (months), median (IQR)**	6.20 [6.03;6.38]	6.1 (6.0,6.5)	6.2 (6.0,6.5)	0.961
**Extracellular volume fraction remote**				
Baseline	27.12 (3.38)	27.02 (3.72)	27.24 (3.04)	
Months 6	27.09 (4.57)	26.45 (3.76)	27.82 (5.37)	
Change	-0.03 (5.23)	-0.56 (4.84)	0.58 (5.74)	
Change difference		-1.15 (-4.58,2.28)		0.502
Change (%)	0.93 (19.27)	-0.84 (17.24)	2.99 (21.73)	
Change difference (%)		-3.84 (-16.48,8.81)		0.542
Adjusted change		-0.43 (5.10)	0.42 (5.50)	
Adjusted change difference		-0.86 (-4.30,2.58)		0.615
**Indexed extracellular volume fraction remote**				
Baseline	16.17 (4.01)	16.26 (3.86)	16.08 (4.27)	
Months 6		13.68 (4.2)	14.73 (3.8)	
Change		-2.57 (3.5)	-1.35 (4.8)	
Change difference		-1.22 (-3.7,1.3)		0.369
Change (%)	1.00 (0.20)	0.98 (0.18)	1.02 (0.22)	
Change difference (%)		-0.04 (-0.17,0.09)		0.591
**Indexed intracellular compartment volume**				
Baseline		44.45 (9.4)	42.96 (10.3)	
Months 6		37.84 (8.4)	38.20 (7.2)	
Change		-6.34 (7.3)	-4.76 (7.4)	
Change difference		-0.94 (-5.1, 3.2)		0.458
Change (%)	1.29 (20.52)	-0.58 (18.65)	3.15 (22.61)	
Change difference (%)		-0.04 (-0.17,0.10)		0.591
**Left ventricular ejection fraction**				
Baseline	41.52 (7.74)	40.63 (5.3)	43.53 (3.9)	
Months 6	47.15 (10.0)	46.95 (10.0)	47.27 (9.9)	
Change	5.63 (10.64)	6.32 (7.7)	3.73 (7.8)	
Change difference		3.71 (-1.5,8.9)		0.158
**Indexed left ventricle end diastolic volume**				
Baseline		74.32 (14.0)	76.38 (19.0)	
Months 6		75.74 (18.2)	77.71 (23.0)	
Change		1.41 (11.4)	1.33 (9.5)	
Change difference		0.22 (-6.7,7.2)		0.949
**Indexed left ventricle end systolic volume**				
Baseline		44.98 (8.7)	42.44 (11.8)	
Months 6		40.14 (12.8)	42.19 (19.0)	
Change		-4.84 (7.5)	-0.25 (10.7)	
Change difference		-5.41 (-11.1,0.3)		0.061
**Stroke volume**				
Baseline	58.13 (15.09)	55.02 (13.77)	61.24 (16.16)	
Months 6	64.57 (22.94)	61.25 (25.95)	68.28 (19.12)	
Change	3.43 (16.43)	3.16 (20.61)	3.70 (11.58)	
Change difference		-0.53 (-13.04,11.97)		0.931
**Indexed left ventricle mass**				

	Total N=39	DAPA N=21	Placebo N=18	p-value
Baseline		63.74 (12.9)	61.99 (14.7)	
Months 6		54.09 (12.6)	55.58 (9.6)	
Change		-9.36 (9.2)	-6.41 (11.1)	
Change difference		-2.23 (-8.1,3.7)		0.446
**Infarct mass**				
Baseline	23.82 (13.67)	22.29 (14.43)	25.60 (12.91)	
Months 6	19.96 (13.73)	18.08 (13.83)	22.15 (13.67)	
Change	-3.86 (10.21)	-4.21 (11.31)	-3.46 (9.07)	
Change difference		-0.76 (-7.48,5.97)		0.821
**Infarct size downstream to the infarct-related artery**				
Baseline	19.80 (12.16)	18.09 (11.48)	21.79 (12.95)	
Months 6	18.77 (12.58)	17.61 (12.79)	20.11 (12.57)	
Change	-1.03 (8.16)	-0.48 (8.96)	-1.68 (7.32)	
Change difference		1.20 (-4.17,6.57)		0.653
**Segments with microvascular obstruction**				
Baseline		1.52 (1.7)	0.89 (1.3)	
Months 6		0.15 (0.4)	0.06 (0.2)	
Change		-1.45 (1.6)	-0.83 (1.2)	
Change difference		0.04 (-0.2,0.2)		0.972

Data are shown as mean (SD)

SD, standard deviation; CMR, cardiac magnetic resonances; IQR, interquartile range

Supplementary Table S12. Changes of cardiac fibrosis markers in the per-protocol population.

	Total<br>N=39	DAPA<br>N=21	Placebo<br>N=18	p
**PICP (pg/mL)**				
Baseline	1200.48 (317.9)	1099.91 (365.7)	1250.51 (292.3)	
Month 6	1061.73 (401.2)	1079.72 (396.5)	1037.46 (416.9)	
Change from baseline	-168.84 (469.8)	-80.87 (487.2)	-213.05 (483.4)	
Change difference		54.35 (-222.9,331.6)		0.693
**P3NP (pg/mL)**				
Baseline	693.55 (354.0)	654.67 (342.4)	726.47 (387.3)	
Month 6	621.56 (365.0)	482.86 (314.3)	718.75 (394.2)	
Change from baseline	-95.23 (506.7)	-222.76 (419.0)	-7.72 (635.9)	
Change difference		-238.84 (-477.6,-0.1)		0.050
**Galectin-3 procollagen (ng/mL)**				
Baseline	1329.83 (894.2)	1202.10 (475.1)	1405.63 (1179.3)	
Month 6	1538.95 (1133.2)	1686.42 (1313.3)	1520.67 (1069.2)	
Change from baseline	191.36 (1505.4)	455.71 (1419.5)	115.04 (1746.3)	
Change difference		135.61 (-676.2,947.4)		0.833
**sST-2 (ng/mL)**				
Baseline	1411.92 (657.0)	1405.84 (720.5)	1367.56 (591.9)	
Month 6	1344.30 (669.5)	1248.97 (764.2)	1470.77 (617.5)	
Change from baseline	-14.63 (773.3)	-30.73 (896.9)	103.21 (697.8)	
Change difference		-202.41 (-669.5,264.7)		0.385
**Troponin I peak (ng/dL)**				
Baseline	166,697.16 (153,693.1)	149,720.8 (133,960.3)	168,221.0 (141,011.0)	
Month 6	22.30 (36.5)	28.72 (51.9)	15.28 (12.1)	
Change from baseline	-161,998.01 (153,693.12)	-132891 (112511.4)	-180113 (155382.7)	
Change difference		17.16 (-11.9,46.2)		0.236
**NT-proBNP peak (pg/mL)**				
Baseline	1501.10 (1075.1)	1413.50 (802.1)	1395.64 (900.3)	
Month 6	448.14 (638.5)	281.19 (212.9)	534.71 (581.5)	
Change from baseline	-1070.47 (638.5)	-1125.33 (767.7)	-793.23 (585.5)	
Change difference		-310.63 (-589.5,-31.7)		0.030

Supplementary Table S13. Sensitivity analysis after adjustment for T2DM and myocardial infarction location.

Variable	Regression estimate	p-value
**Extracellular volume fraction remote**		
Change difference	-1.66 (-4.73,1.41)	0.281
Change difference (%)	-6.71 (-18.45,5.03)	0.255
**Indexed extracellular volume fraction remote**		
Change difference	-0.91 (-3.62,1.81)	0.503
Change difference (%)	-0.07 (-0.19,0.06)	0.290
**Indexed intracellular compartment volume**		
Change difference	0.82 (-1.14,2.77)	0.402
Change difference (%)	-0.07 (-0.20,0.06)	0.289
**Left ventricular ejection fraction**		
Change difference	4.51 (-1.52,10.55)	0.139
**Indexed left ventricle end diastolic volume**		
Change difference	0.06 (-6.39,6.28)	0.986
**Indexed left ventricle end systolic volume**		
Change difference	-4.92 (-9.75,-0.09)	0.047
**Stroke volume**		
Change difference	-0.53 (-13.04,11.97)	0.931
**Left ventricle mass**		
Change difference	-4.58 (-16.57,7.42)	0.443
**Infarct mass**		
Change difference	2.13 (-4.37,8.62)	0.512
**Infarct size downstream to the infarct-related artery**		
Change difference	2.73 (-2.27,7.73)	0.276
**Segments with microvascular obstruction**		
Change difference	-0.65 (-1.60, 0.30)	0.176

Data are shown as mean (SD)

SD, standard deviation; CMR, cardiac magnetic resonances; IQR, interquartile range

SUPPLEMENTARY FIGURES

Supplementary Figure S1. Subgroup analyses.

Extracellular volume fraction remote

Subgroup	N	Dapagliflozin	Placebo	Difference (95% CI)	Pint
**Sex**					
Male	44	-0.17 (4.7)	1.45 (5.9)	-1.62 (-4.8, 1.6)	-
Female	3	-3.00 (4.2)	1.00 (-)	-	
**Age**					
≥60	21	-0.98 (5.8)	2.03 (5.2)	-3.01 (-8.0, 2.0)	0.479
<60	26	-0.02 (4.0)	0.77 (6.5)	-0.79 (-5.0, 3.4)	
**Diabetes mellitus**					
Yes	4	-5.75 (6.0)	4.83 (1.6)	-10.58 (-29.5, 8.5)	0.078
No	43	0.06 (4.4)	1.07 (5.9)	-1.01 (-4.2, 2.2)	
**Myocardial infarction location**					
Anterior	36	0.54 (3.8)	1.49 (5.9)	-0.96 (-4.3, 2.4)	0.398
Non-anterior	11	-2.90 (6.1)	1.17 (5.6)	-4.07 (-12.5, 4.4)	
**Chronic kidney disease**					
Yes	5	-0.11 (3.4)	8.20 (9.7)	-8.27 (-26.3, 9.8)	0.149
No	42	-0.43 (4.9)	0.72 (5.1)	-1.15 (-4.2, 2.0)	
**Baseline LVEF**					
<40%	12	2.61 (3.8)	4.94 (8.7)	-2.33 (-10.0, 5.4)	0.894
40-50%	35	-1.98 (4.3)	0.84 (5.2)	-2.82 (-6.1, 0.5)	
**Infarct size**					
Larger	23	1.80 (4.1)	1.61 (6.4)	0.19 (-4.5, 4.9)	0.267
Smaller	24	-2.00 (4.5)	1.19 (5.1)	-3.18 (-7.3, 0.9)	
**NT-proBNP**					
Higher	20	1.37 (3.8)	3.06 (7.1)	-1.69 (-7.2, 3.8)	0.885
Lower	19	-1.47 (4.4)	0.28 (3.5)	-1.20 (-5.5, 3.2)	
**PICP**					
Higher	25	-1.56 (5.4)	1.65 (7.1)	-3.22 (-8.5, 2.0)	0.376
Lower	22	0.62 (3.8)	1.06 (2.6)	-0.44 (-3.6, 2.7)	
**PIIINP**					
Higher	24	0.13 (4.0)	2.09 (6.1)	-1.96 (-6.3, 2.3)	0.909
Lower	23	-0.91 (5.3)	0.70 (5.5)	-1.61 (-6.3, 3.1)	
**GAL-3**					
Higher	24	0.44 (4.9)	3.35 (3.6)	-3.79 (-7.5, -0.1)	0.132
Lower	23	0.35 (4.6)	-1.13 (7.1)	0.78 (-4.3, 5.8)	
**sST-2**					
Higher	23	1.14 (5.3)	4.67 (5.1)	-3.53 (-8.0, 1.0)	0.148
Lower	24	-1.70 (3.8)	-2.13 (4.1)	0.43 (-2.9, 3.8)	
**Microvascular obstruction**					
Yes	22	1.18 (4.1)	0.28 (5.1)	0.90 (-3.2, 5.0)	0.095
No	25	-1.74 (4.8)	2.47 (6.3)	-4.21 (-8.8, 0.4)	
**ACEi/ARBs/ARNI**					
Yes	10	-1.58 (6.5)	4.81 (5.5)	-6.39 (-15.2, 2.4)	0.101
No	37	-0.17 (4.4)	0.08 (5.4)	-0.25 (-3.5, 3.0)	
**BMI**					
≥25 Kg/m²	32	-0.89 (4.9)	1.51 (5.4)	-2.40 (-6.2, 1.4)	0.535
<25 Kg/m²	15	0.95 (3.8)	1.29 (6.6)	-0.34 (-6.5, 5.8)	
**LVEDVi**					
Higher	23	1.14 (5.3)	4.67 (5.1)	-3.52 (-8.0, 1.0)	0.306
Lower	24	-1.70 (3.8)	-2.13 (4.1)	0.43 (-2.9, 3.8)	
**LVESVi**					
Higher	23	0.68 (4.1)	3.52 (6.2)	-2.84 (-7.2, 1.5)	0.843
Lower	24	-2.10 (5.2)	0.14 (5.2)	-2.24 (-6.8, 2.3)	
**LVMi**					
Higher	20	-0.14 (4.5)	0.34 (5.1)	-0.49 (-4.6, 3.6)	0.402
Lower	27	-0.60 (5.3)	2.62 (6.4)	-3.22 (-8.8, 2.3)	
**tCRM**					
Higher	24	0.60 (4.5)	1.12 (5.1)	-0.52 (-4.6, 3.5)	0.394
Lower	23	-1.38 (4.8)	1.77 (6.6)	-3.15 (-8.1, 1.8)	

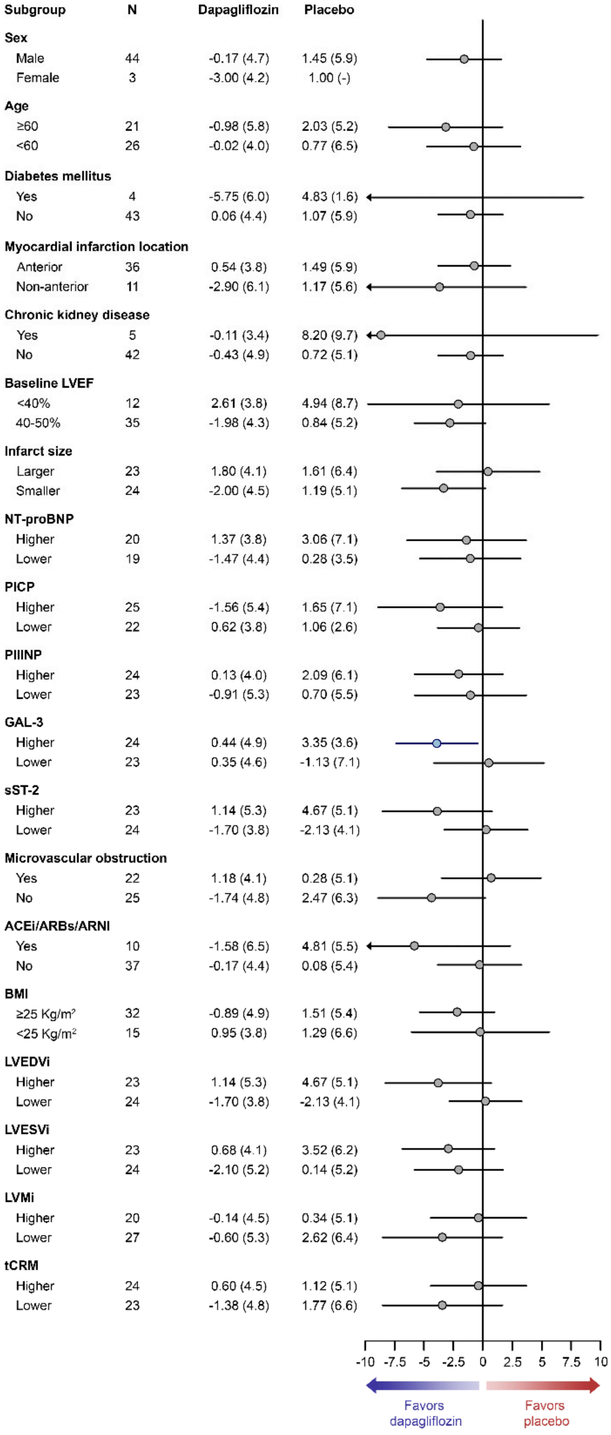

References:

11. Ibanez, B.; James, S.; Agewall, S.; Antunes, M.J.; Bucciarelli-Ducci, C.; Bueno, H.; Caforio, A.L.P.; Crea, F.; Goudevenos, J.A.; Halvorsen, S.; et al. 2017 Esc Guidelines for the Management of Acute Myocardial Infarction in Patients Presenting with St-Segment Elevation: The Task Force for the Management of Acute Myocardial Infarction in Patients Presenting with St-Segment Elevation of the European Society of Cardiology (Esc). *Eur. Heart J.* **2018**, *39*, 119–177. https://doi.org/10.1093/eurheartj/ehx393.
